# Lumican Accelerates Wound Healing by Enhancing α2β1 Integrin-Mediated Fibroblast Contractility

**DOI:** 10.1371/journal.pone.0067124

**Published:** 2013-06-26

**Authors:** Xiao-Jin Liu, Fan-Zhi Kong, Ya-Hui Wang, Jiang-Hong Zheng, Wei-Dong Wan, Chen-Liang Deng, Guang-Yu Mao, Jun Li, Xiao-Mei Yang, Yan-Li Zhang, Xue-li Zhang, Song-lin Yang, Zhi-Gang Zhang

**Affiliations:** 1 Department of Plastic Surgery, Shanghai Jiao Tong University Affiliated Sixth People's Hospital, Shanghai, China; 2 State Key Laboratory of Oncogenes and Related Genes, Shanghai Cancer Institute, Ren Ji Hospital, Shanghai Jiao Tong University School of Medicine, Shanghai, China; 3 Department of General Surgery, Central Hospital of Fengxian District, Shanghai, China; The University of Hong Kong, Hong Kong

## Abstract

Lumican is a dermatan sulfate proteoglycan highly expressed in connective tissue and has the ability to regulate collagen fibril assembly. Previous studies have shown that lumican is involved in wound healing, but the precise effects of lumican on reepithelialization and wound contraction, the two pivotal aspects of skin wound healing, have not been investigated. Here we explored the roles of lumican in fibroblast contractility, a main aspect of skin wound healing, by adopting mice skin wound healing model and the corresponding *in vitro* cellular experiments. Our results showed that lumican can promote skin wound healing by facilitating wound fibroblast activation and contraction but not by promoting keratinocyte proliferation and migration. Silencing of integrin α2 completely abolished the pro-contractility of lumican, indicating lumican enhances fibroblast contractility via integrin α2. Our study for the first time demonstrated that lumican can affect fibroblast’s mechanical property, which is pivotal for many important pathological processes, such as wound healing, fibrosis, and tumor development, suggesting that lumican might have a potential to be used to modulate these processes.

## Introduction

Wound healing is a complex and dynamic pathological process, finely regulated by intracellular signaling and extracellular components, including cytokines, growth factors, extracellular matrix (ECM) proteins, and so on [1], [2].

Accumulating evidences have shown that ECM proteins play important roles in skin cells proliferation, differentiation or motility during wound healing process. Knockdown of osteopontin promotes wound repair and reduces scarring by reducing leukocyte recruitment and altering ECM deposition and angiogenesis [3]. Wound healing is delayed in sydecan-4 null mice, which is closely associated with reduced granulation tissue and wound contraction in sydecan-4 null mice compared to that in wild type littermates [4]. Dermatopontin, a tyrosine-rich ECM protein with a striking tendency to bind the small dermatan sulfate proteoglycan, can promote wound healing by affecting migration and proliferation of local cells [5].

Lumican, a small dermatan sulfate proteoglycan, is highly expressed in interstitial tissue [6] and can modulate collagen fibril organization [7], inflammatory cells migration, infiltration [8], [9] and keratinocyte phenotypes [7], [8], [9], [10]. Nevertheless, there is no report about the effects of lumican on fibroblast, one of the major cell types in wound healing. Corneal epithelial wound healing was retarded by knockout of lumican or anti-lumican antibody [11], while it was promoted by purified lumican protein [10]. Further investigation revealed that lumican is also required for skin wound repair by showing that skin wound healing is significantly delayed in lumican null mice compared to that in wild type mice [7]. Although the importance of lumican in wound healing had been established, the mechanisms of how lumican affects wound healing, particularly fibroblast behaviors, has not been studied yet.

In this study, we investigated the underlying mechanisms of how lumican affects wound healing. We first applied purified lumican protein to the mice skin wound model and found that lumican promoted mice skin wound healing. Second, we revealed that recombinant lumican enhanced fibroblasts activation but not epidermis migration or skin cells proliferation *in vivo* by using immunohistochemistry analyses, and these results are consistent with our *in vitro* cellular function assays, which showed that recombinant lumican enhanced the contractility of fibroblasts in three-dimensional surroundings, but neither the migration of keratinocytes nor the proliferation of these two cell types. Further analyses revealed that silencing of integrin α2 but not integrin α1 inhibited the contractility promoting ability of lumican, indicating that effects of lumican on fibroblast contractility in wound healing is mainly mediated by integrin α2.

## Materials and Methods

### Lumican Recombinant Protein and Lumican Core Protein Expression, Purification and Characterization

Lumican ORF was cloned into the episomal expression vector V152 with pCEP-Pu-Strep II-tag (C-terminal) in-frame and the sequence of the BM-40 (SPARC/osteonectin) signal peptide downstream of the CMV promoter. Lumican was recombinant expressed in EBNA-293 cells after transfecting reconstructed plasmid by using X-tremeGENE 9 DNA Transfection Reagent (Roche, Mannheim, Germany). 2 days after transfection, the cells were screening with 2 µg/ml puromycin (Sigma-Aldrich, St. Louis, MO) in Dulbecco’s modified Eagle’s medium (DMEM, 11965, Gibco, Grand Island, NY) supplemented with 10% fetal bovine serum (FBS, Gibco-BRL, Gaithersburg, MD) for 7 days, then the culture media was collected and applied to the Strep Tactin sepharose column (IBA, Gottingen, Germany). After this, the column was washed with binding buffer and eluted by elution buffer containing 2.5 mM desthiobiotin. The collected fractions were further quantified by a Nanodrop 2000 spectrophotometer (Thermo Fisher Scientific, Wilmington, DE) and BCA Protein Assay Kit (Pierce. Biotechnology Inc, Rockford, IL) and identified by western blot assay.

Lumican core protein was expressed and purified as previously described [12]. The collected core protein was identified by western blot assay, the concentration of core protein was measured by a Nanodrop 2000 spectrophotometer and BCA Protein Assay Kit. The purity of lumican recombinant protein and lumican core protein was determined by Coomassie brilliant blue staining.

### Deglycosylation of Lumican Recombinant Protein

Deglycosylation of lumican recombinant protein was conducted by using N-glycosidase F (New England Biolabs, Beverly, MA) according to the manufacturer’s instructions.

### Mice Wound Healing Model

Six-week-old male Balb/c mice were purchased from the Shanghai Slac Laboratory Animal CO. LTD (Slac, Shanghai, China). Mice wound healing experiment was conducted in Shanghai Jiao Tong University. All procedures were approved by the Shanghai Jiao Tong University Animal Care and Use Committee; all animals were housed under standard conditions under institution-approved guidelines.

Briefly, after anesthesia, the dorsal hair of mice was shaved, and a 5 mm round full-thickness skin excision wound was made at midline of the back 2-cm caudal ward from the skull of each mouse. Lumican recombinant protein (20 nM) or adjuvant (elution buffer with 30% glycerol) was topical applied to wound from post-injury day 0 to day 7 once daily. 30 mice were divided into two groups, one group was treated with lumican, and the other group was treated with adjuvant as control. Wound area was measured at post-injury day 0, 3, 7, 10 and 14. At post-injury day 3, 7 and 14, wound tissues of 5 mice of each group were harvested for further analyses. 10 mice were used to investigated the roles of lumican core protein in dorsal skin wound healing. Lumican core protein (20 nM) or adjuvant (elution buffer with 30% glycerol) was applied to wound from post-injury day 0 to day 7 once daily, Wound area was measured at post-injury day 0, 3, 7, 10 and 14.

### Hematoxylin and Eosin (HE) and Immunohistochemistry Stain

Paraformaldehyde-fixed, paraffin-embedded tissue sections (5 µm) at the midlines of wound specimen were adopted for HE and immunohistochemistry stain. For immunohistochemistry stain, the sections were detected with primary antibodies against proliferating cell nuclear antigen (PCNA, Epitomics, Burlingame, CA), α-smooth muscle actin (α-SMA, Sigma-Aldrich, St Louis, MO) overnight at 4°C. After incubated with appropriate secondary antibodies, the sections were developed with diaminobenzidine and counterstained with haematoxylin. Images were processed for publication using Adobe Photoshop CS4.

### Cell Culture

Immortal human keratinocytes (HaCaT) (China Center for Type Culture Collection, Wuhan, China) [13], primary human dermal fibroblasts (HDF) and human embryonic kidney 293/Epstein-Barr nuclear antigen cells (EBNA-293) were maintained in DMEM supplemented with 10% FBS, 100 U/ml penicillin and 100 µg/ml streptomycin (Solarbio, Beijing, China). HDF cells were obtained from adolescent foreskin tissue of patients of Shanghai Jiao Tong University affiliated Sixth People's Hospital with written informed consent signed by their parents or guardians on the behalf of the participants according to Declaration of Helsinki Principles. All cellular experiments were conducted in Shanghai Jiao Tong University. The study was approved by the Ethics Committee of Shanghai Jiao Tong University affiliated Sixth People's Hospital. All cells were incubated at 37°C humidified atmosphere with 5% CO2. HDF at passage 4–8 were adopted for experiments.

### Cell Proliferation Assay

Proliferation was determined by using a standard CCK-8 cell counting kit (Dojindo, Kumamoto, Japan) according to the manufacturer’s instruction. HaCaT (2.0×10^4^ cells/ml) and HDF cells (4.0×10^4^ cells/ml) were seeded in 96-well plates (100 µl/well). The medium was replaced 24 hours later, and lumican recombinant protein (0, 0.1 nM, 1 nM, 10 nM) was added in. The medium was changed once every two days and optical density (OD_450_) values were measured at day 0, 2, 4 and 6 respectively by a microplate reader (SpectraMax M5, Molecular device).

### Scratch Wound Healing Assay

HaCaT cells at 3×10^5^/ml were seeded in 24-well plates (500 µl/well) and cultured to confluence as a monolayer, and starved by DMEM supplied with 1% FBS for 24 hours, then gently scratch the monolayer across the centre of the cell with a 10 µl pipette tip. Wash with PBS for 3 times and changed by DMEM supplied with 1% FBS and different concentration of lumican recombinant protein (0, 0.1 nM, 1 nM, 10 nM). The gaps were photographed every 6 hours until 24 hours post-scratch by a live cell imaging system (Olympus, Tokyo, Japan).

### Fibroblast Activation Assay

1×10^4^/well HDF cells were seeded in 24-well plates with serum-free DMEM medium, and lumican recombinant protein (10 nM) or adjuvant control was added in to treat for 3 days. Then cells were immunofluorescence stained as described [14]. DAPI (Sigma-Aldrich, St Louis, MO) was used to stain cell nucleus, and α-SMA primary antibody (Sigma-Aldrich, St Louis, MO) was used to stain α-SMA. The percentage of activated fibroblasts were calculated as α-SMA positive cells divided DAPI positive cells in 5 random fields of each well.

### 3D Collagen Gel Contraction Assay

3D collagen gel contraction assays were conducted as reported [14]. Lumican recombinant protein at 0, 0.1 nM, 1 nM or 10 nM was added into the collagen gel lattices.

For activated myofibroblasts gel contraction assay, fibroblasts were activated by TGF-β1 (2 ng/ml) for 5 days, then collected and used.

### SiRNA Transfection

SiRNA duplexes targeted at integrin α1 or α2 and scramble control siRNA duplex were obtained from GenePharma (Shanghai, China). Transfection was performed by using the Lipofectamine RNAiMAX Reagent (Invitrogen, Carlsbad, CA) according to the manufacturer’s instruction.

### Western Blot Assay

The primary antibodies against integrin α1 (abcam, Cambridge, MA), integrin α2 (abcam, Cambridge, MA) and GAPDH (abcam, Cambridge, MA) were incubated overnight at 4°C. Signals on the membranes were detected by an Odyssey infrared imaging system (LI-COR, Lincoln, NE) after incubating with the IRDye 800 anti-rabbit or IRDye 680 anti-mouse (LI-COR, Lincoln, NE) secondary antibodies for 1 hour at room temperature.

### Statistical Analysis

Statistic differences were calculated using two-tailed Student’s t test. *p*<0.05 was considered statistically significant.

## Results

### Lumican and Lumican Core Protein Promotes Mice Skin Wound Healing

**Figure 1:**
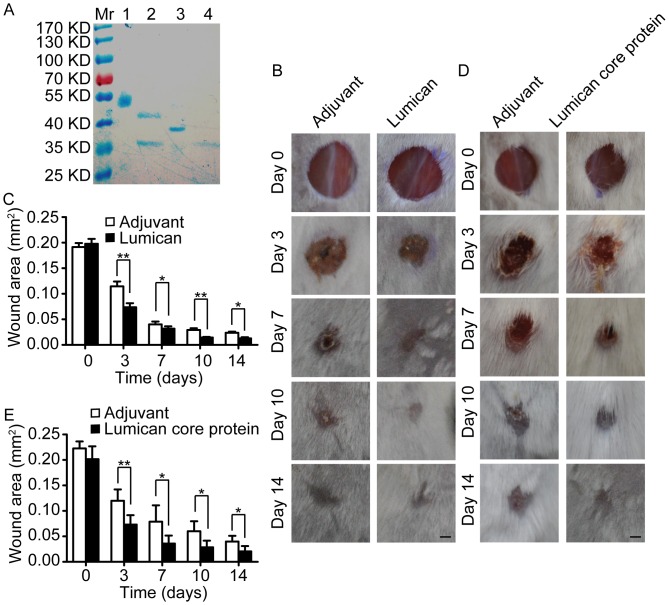
Lumican and lumican core protein promotes mice skin wound healing. (A) Coomassie brilliant blue staining of lumican protein. Lane 1 is glycosylated lumican recombinant protein with Strep-II tag expressed in EBNA-293 cells. Lane 2 is deglycosylated lumican protein after digested by N-glycosidase F. Lane 3 is lumican core protein with His tag expressed in prokaryotic system. Lane 4 is N-glycosidase F. The molecular weight (MW) of N-glycosidase F is 35 KD, the MW of Strep-II tag is 3.7 KD, and His tag is 1 KD. So the MW of deglycosylated lumican protein is about 42 KD, and the MW of lumican core protein is 39 KD. Mr: molecular mass ladder. (B) Representative images of glycosylated lumican protein treated mice skin wound. Scale bar = 1 mm. (C) Quantification of glycosylated lumican protein treated mice wound area. Data are presented as means±SD, n = 5, *p<0.05, **p<0.01. (D) Representative images of lumican core protein treated mice skin wound. Scale bar = 1 mm. (E) Quantification of lumican core protein treated mice wound area. Data are presented as means±SD, n = 5, *p<0.05, **p<0.01.

To investigate the *in vivo* roles of lumican in skin wound healing as a secreted protein, we purified recombinant lumican protein and lumican core protein, then applied them to a mice full-thickness skin excision wound model. After 7 days of daily topical administration, we found that recombinant glycosylated lumican protein and lumican core protein can both significantly promoted mice skin wound healing. As illustrated in figure 1A, lumican recombinant protein expressed by EBNA-293 cells is glycosylated, and can be deglycosylated by N-glycosidase F [12]. The wound area of lumican or lumican core protein-treated mice was obviously smaller than that of adjuvant-treated mice from post-injury day 3 to day 14 (Figure 1B–E). Because the adjuvant for lumican recombinant protein and lumican core protein is different, we didn’t compare the efficiency between these two kind of proteins. The general condition was not different between these groups of mice (data not shown).

The result indicates that the wound healing promoting effect of lumican is at least not totally dependent on the dermatan sulfate chain.

### Recombinant Lumican Protein does not Significantly Affect Activation of Keratinocytes

Skin wound healing is coordinately accomplished by two major events, wound contraction and reepithelialization [15]. Wound contraction is primarily generated by fibroblast activation and contraction, while reepithelialization is a process constructed by migration and proliferation of keratinocyte [16], which is also termed as keratinocyte activation [17], [18].

**Figure 2:**
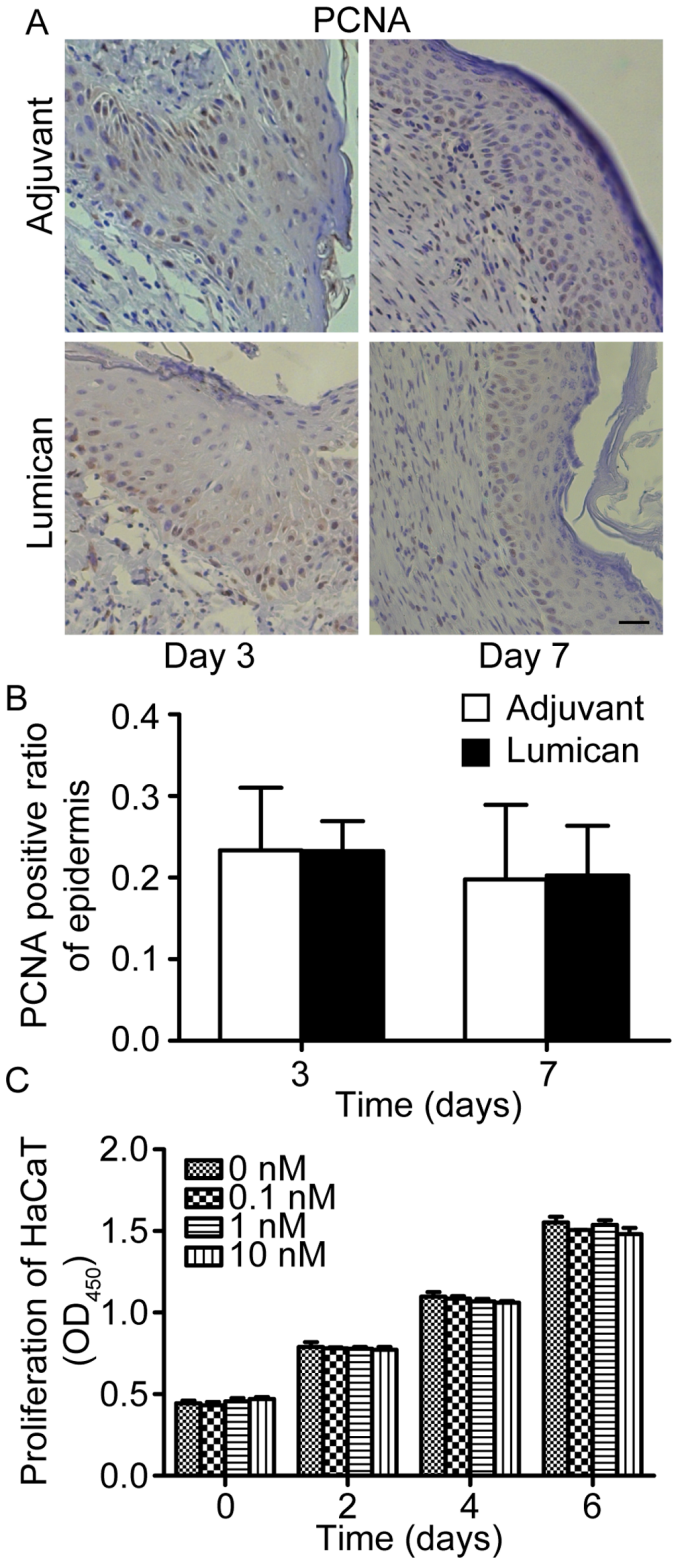
Recombinant lumican protein did not significantly influence keratinocyte proliferation. (A) Images of PCNA stained mice wound sections at post-injury day 3 and day 7. Scale bar = 25 µm. (B) Quantification of PCNA positive cells in epidermis. Values represent means±SD, n = 5. (C) Results of *in vitro* CCK-8 HaCaT cells proliferation assays. Values are denoted as means±SEM, n = 3.

To explore the mechanism of how lumican affects wound healing, we first evaluated the roles of recombinant lumican protein in keratinocyte proliferation *in vivo* and *in vitro*. No obvious difference was observed on PCNA staining in wound epidermis between lumican-treated mice and adjuvant-treated mice (Figure 2A and 2B). And *in vitro* CCK-8 proliferation assay also displayed that recombinant lumican protein did not affect HaCaT cells proliferation (Figure 2C).

**Figure 3:**
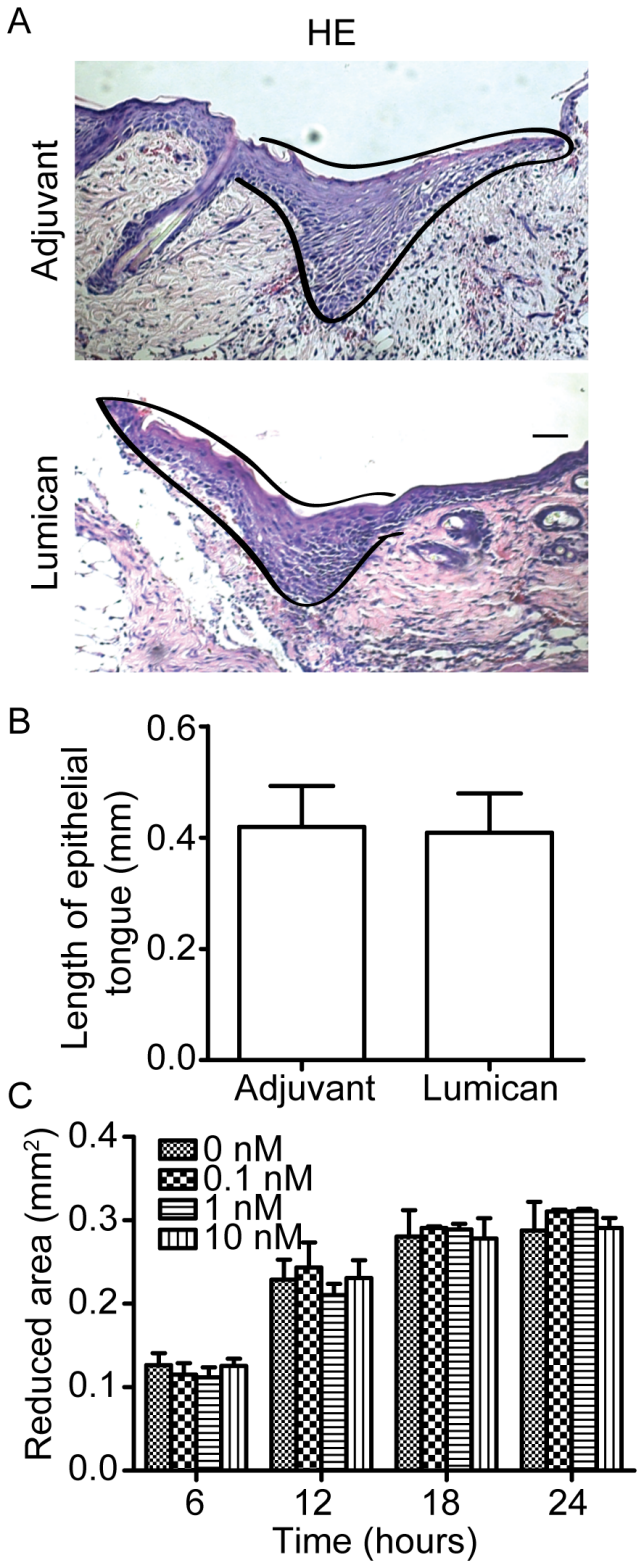
Recombinant lumican protein did not affect keratinocyte migration statistically. (A) Epidermal tongue in HE stained mice wound sections at post-injury day 3. The black lines outline the epidermal tongue. Scale bar = 50 µm. (B) Quantification of epidermal tongue length. Values are means±SD, n = 5. (C) Quantification of *in vitro* HaCaT cells scratch wound healing assays after treating with lumican at 0, 0.1, 1 and 10 nM. Values are means±SEM, n = 3.

Then *in vivo* migration of epidermal cells was evaluated by measuring the length of epidermal tongue in wound sections. The results showed that lumican had no effect in the length of epidermal tongue *in vivo* (Figure 3A and 3B). Scratch wound healing assays were also conducted to examine the effect of lumican on HaCaT cell migration *in vitro*, the results displayed no significant difference between lumican-treated and adjuvant-treated cells (Figure 3C).

Together, these data indicate that recombinant lumican protein has no effect on keratinocyte activation both *in vivo* and *in vitro*.

### Recombinant Lumican Protein Promotes Fibroblast Activation*in vivo* and *in vitro*

**Figure 4:**
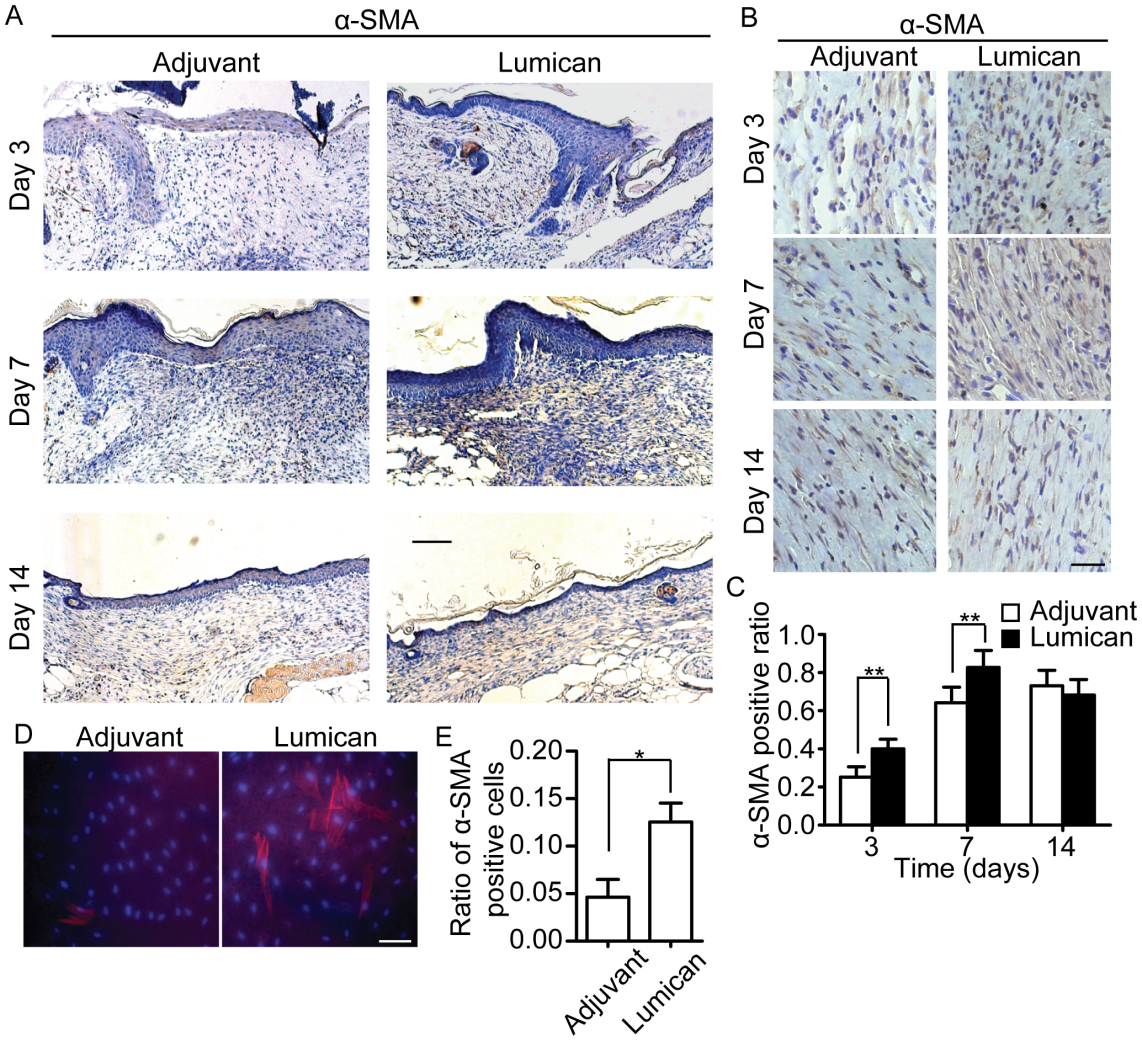
Lumican activated fibroblasts in vivo and in vitro. (A) Low magnification images of α-SMA stained mice wound tissue. Scale bar = 100 µm. (B) High magnification images of α-SMA stained mice wound tissue. Scale bar = 25 µm. (C) Quantification of α-SMA positive cells ratio. Values are denoted as means±SD, n = 5, **p<0.01. (D) Immunofluorescence staining of α-SMA. α-SMA is shown by red fluorescence and cell nucleus is stained by DAPI. Scale bar = 25 µm. (E) Ratio of α-SMA positive HDF cells. Values means±SEM, n = 3. *p<0.05.

We further examined the effect of lumican on fibroblast behaviors *in vivo* and *in vitro*. α-SMA, a fibroblast activation marker, was immunostained in lumican-treated or adjuvant treated mice wound sections. The results showed that the ratio of α-SMA positive stromal cells in wound granulation tissue of lumican-treated mice was significantly higher than that of adjuvant-treated mice at 3 and 7 days post-injury, but no difference was observed at 14 days post-injury (Figure 4A–4C). The results indicate that topical usage of recombinant lumican protein can promote fibroblast activation in the early wound healing process, while this effect would not last to later proliferation stage when activated fibroblasts would contribute to excessive scar formation.

We also investigated the *in vitro* effects of lumican on fibroblasts activation. As displayed in figure 4D and 4E, the ratio of α-SMA positive cells of lumican treated HDF was significantly higher than that of adjuvant treated HDF. That means lumican can also activate HDF cells *in vitro*.

**Figure 5:**
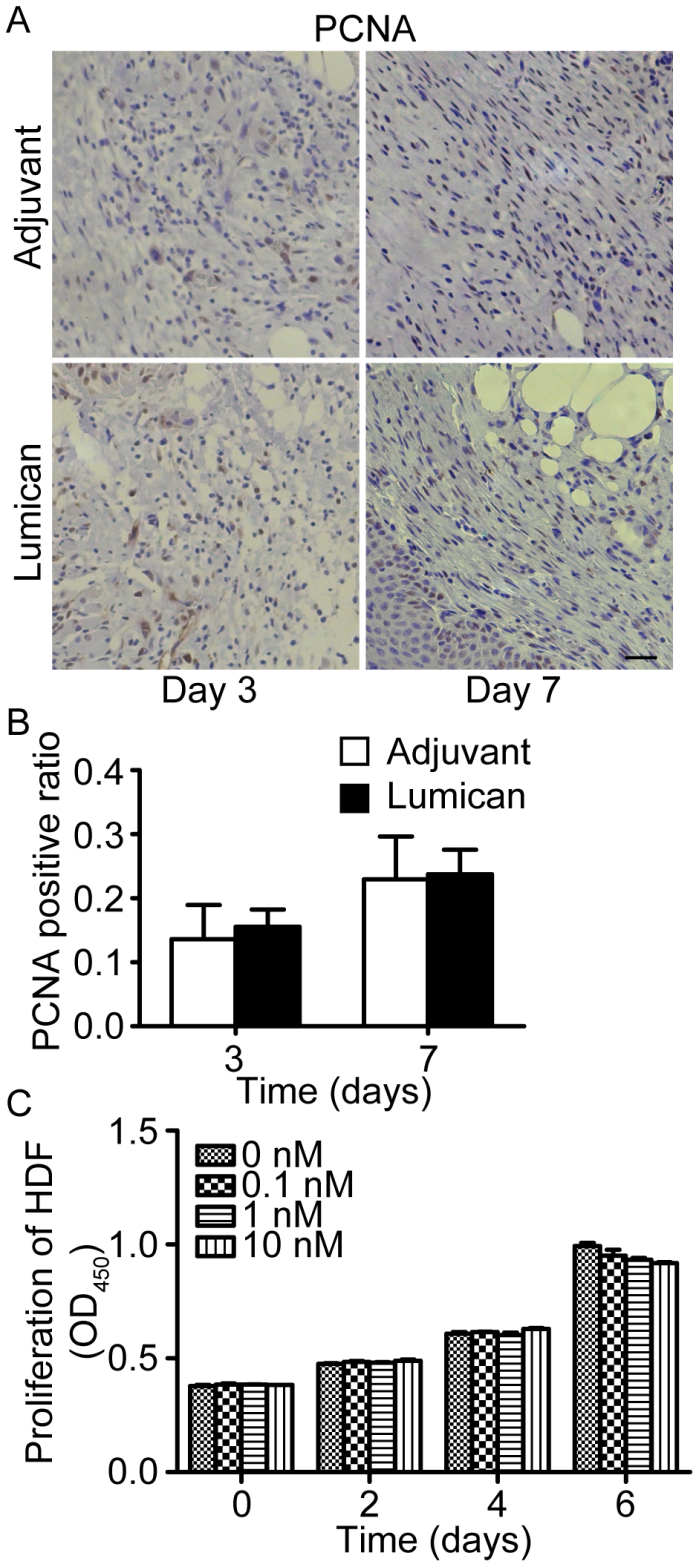
Fibroblast proliferation was not affected by lumican. (A) Images of PCNA stained mice wound granulation tissue at post-injury day 3 and day 7. Scale bar = 25 µm. (B) Quantification of PCNA positive cells in granulation tissue. Values represent means±SD, n = 5. (C) Results of *in vitro* CCK-8 HDF cells proliferation assays. Values are denoted as means±SEM, n = 3.

Besides fibroblast activation, fibroblast proliferation is also involved in wound repair. We further investigated the effects of lumican on fibroblast proliferation *in vivo* and *in vitro*. Evaluated by PCNA staining, the stromal cell proliferation in wound granulation tissue was similar between lumican-treated mice and adjuvant-treated mice at 3 and 7 days post-injury (Figure 5A and 5B). And *in vitro* CCK-8 proliferation assay also showed that lumican did not promote or inhibit fibroblast proliferation (Figure 5C).

### Lumican Enhances Fibroblast Contractility in Three-dimensional Surroundings

**Figure 6:**
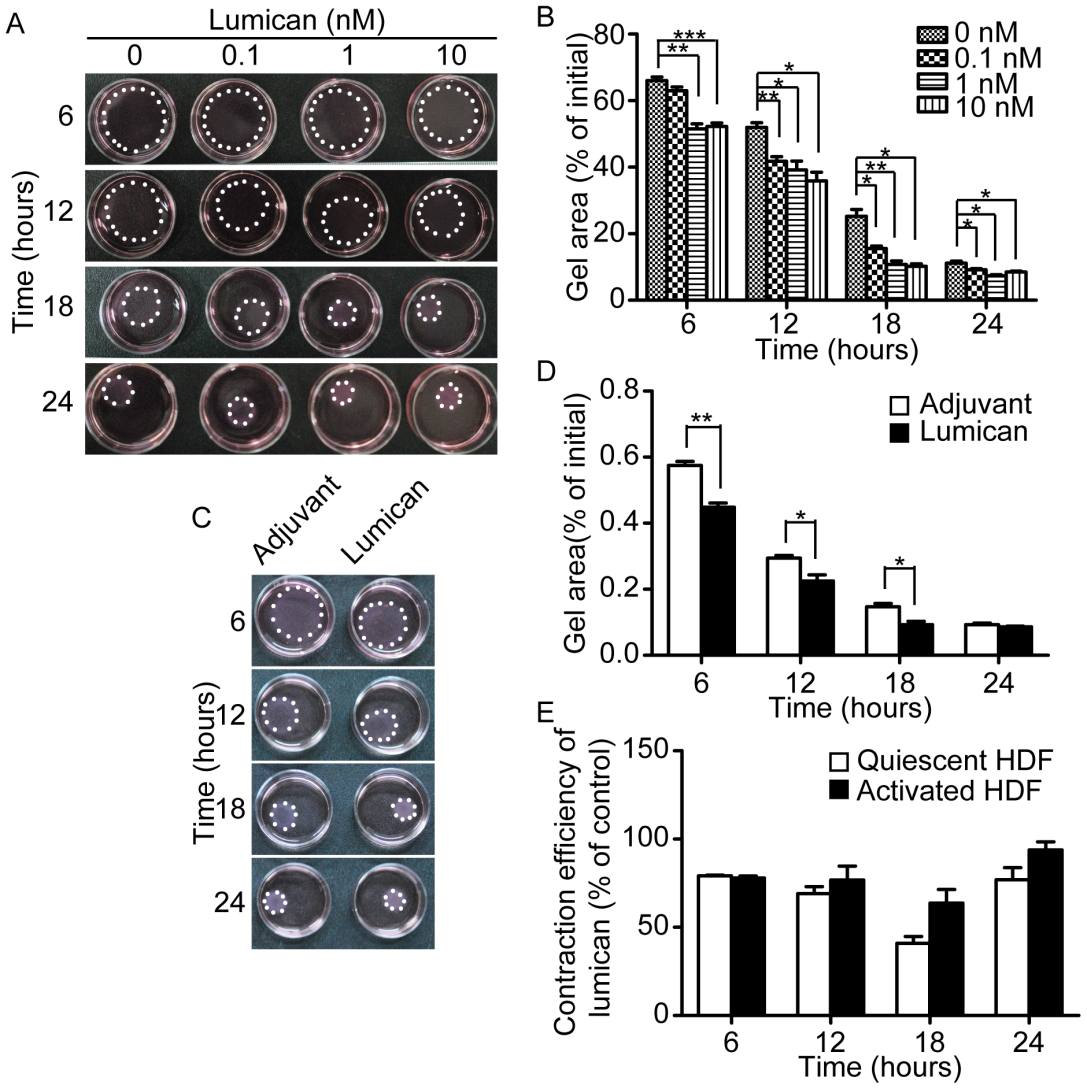
Lumican enhanced contraction of fibroblasts-populated collagen gel lattices. (A) Representative images of quiescent fibroblasts populated collagen gel lattices at different time points. The gels are outlined by white spots. (B) Quantification of quiescent fibroblasts populated collagen gel areas. Values are means±SEM, n = 3, *p<0.05, **p<0.01, ***p<0.001. (C) Representative images of activated fibroblasts populated collagen gel lattices at different time points. The gels are outlined by white spots. Lumican used in the assay is 10 nM. (D) Quantification of activated fibroblasts populated collagen gel areas. Values are means±SEM, n = 3, *p<0.05, **p<0.01. (E) Comparison of contraction efficiency of lumican (10 nM) on quiescent or activated HDF.

Because activated fibroblast is defined not only by α-SMA expression, but also by higher contractility [19]. To examine the effect of lumican on fibroblast contractility, three-dimensional collagen gel contraction assay was performed. The results showed that lumican can enhance contractility of fibroblast in a dose-dependent manner, further confirming our *in vivo* finding that lumican can promote fibroblast activation (Figure 6A and 6B).

Besides in quiescent fibroblasts, we also tested the pro-contractile ability of lumican on TGF-β1 activated myofibroblasts. After activation with TGF-β1 (2 ng/ml) for 5 days, HDF cells were collected and used. The result showed that lumican can also enhance the contractile ability of activated fibroblasts (Figure 6C and 6D), and the pro-contractility effect of lumican in quiescent fibroblasts and activated fibroblasts is not significantly different (Figure 6E).

Taken together, these results demonstrate that recombinant lumican protein facilitates wound healing by promoting fibroblast activation but not by accelerating wound reepithelialization.

### Silencing of Integrinα2 Blocks the Pro-contractility Effect of Recombinant Lumican Protein

Fibroblast contraction requires participation of ECM, focal adhesion and cytoskeleton [20], while integrins are the core of focal adhesion, which mediate the connection between ECM and cytoskeleton [21], [22]. Integrin α1 and α2 are two highly expressed integrins in fibroblasts that recognize collagen [23]. Previous studies had revealed that lumican can interact with integrin α2 in endothelial cells [24] and melanoma cells [25]. So we presumed that lumican might enhance fibroblast contractility via integrin α1β1 or α2β1.

**Figure 7:**
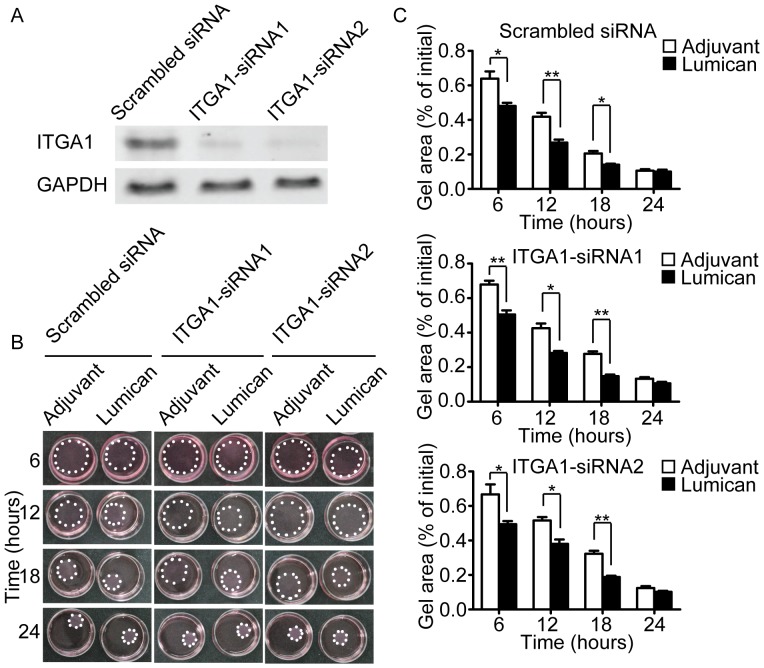
Silencing of integrinα1 did not block the pro-contractility of lumican. (A) Silencing efficiency of integrin α1 was evaluated by western blotting. (B) Representative images of contracted collagen gel lattices at different time points. Lumican used in the experiments was 10 nM. The gels are outlined by white spots. (C) Quantification of gel areas. Values are means±SEM, n = 3, *p<0.05, **p<0.01.

**Figure 8:**
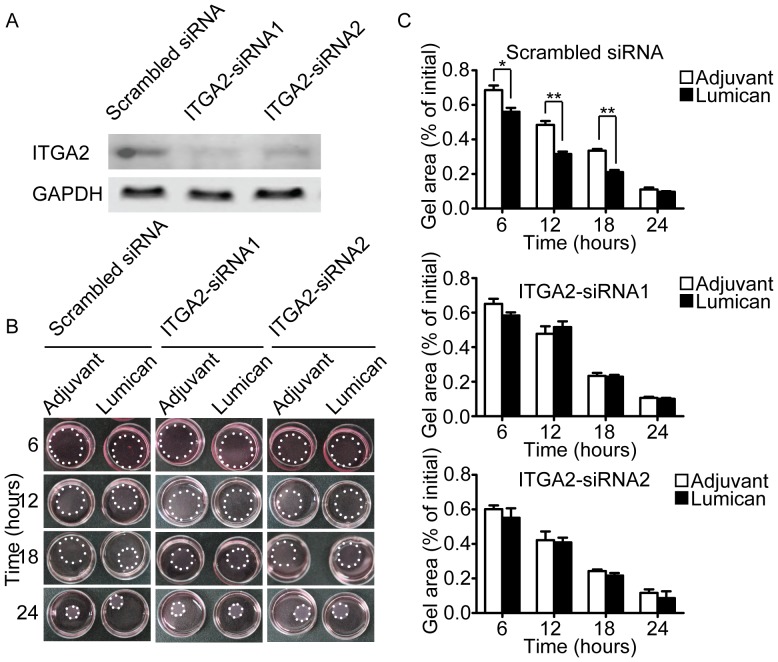
Silencing of integrinα2 abolished the pro-contractility of lumican. (A) Silencing efficiency of integrin α2 was evaluated by western blotting. (B) Representative images of contracted collagen gel lattices at different time points. Lumican used in the experiments was 10 nM. The gels are outlined by white spots. (C) Quantification of gel areas. Values are means±SEM, n = 3, *p<0.05, **p<0.01.

To verify this hypothesis, we conducted fibroblast collgen lattice contraction assay after silencing of integrin α1 or α2. The results showed that silencing of integrin α1 didn’t block the pro-contractility of lumican in three-dimensional condition (Figure 7A–C), but silencing of integrin α2 completely abolished the pro-contractility of lumican (Figure 8A–C), clearly indicates that the pro-contractile effect of lumican in fibroblast is mainly dependent on integrin α2.

## Discussion

Lumican is a highly-expressed secreted protein in connective tissue and has the ability to regulate collagen fibril organization [7]. It has been demonstrated that lumican is involved in inflammatory cells infiltration [7] and angiogenesis [24], the two substantial aspects of wound healing. Lumican was also suggested to be involved in reepithelialization in corneal wound model. However, the roles of lumican in wound contraction and epidermal coverage have not been explored in skin wound yet. Our study explored these two aspects and demonstrated that lumican can facilitate skin wound healing by enhancing fibroblast contractility via integrin α2, which deepens our understanding about the roles of lumican in interstitial tissue by displaying that lumican can modulate activation of fibroblast, the major stromal cell in connective tissue.

To the best of our knowledge, this is the first report about the roles of lumican on fibroblast behaviors. Fibroblast plays a fundamental role in wound healing by secreting chemokines, growth factors and ECM proteins that is necessary for inflammatory cells infiltration and neoangiogenesis [15]. Activated fibroblasts express α-SMA, which upregulates the contractility of fibroblasts [19], thus promote wound contraction by exerting higher contractile force to wound edges [26], [27], [28]. Wound contraction can shrink the size of wound, thus facilitates wound repair, so it is a crucial event in wound healing process [29]. And 3D collagen gel contraction assay simulates the 3D collagen-riched environment of wound granulation tissue, so it is a useful in vitro model for studying wound contraction [30]. It is difficult to quantitatively measure wound contraction *in vivo*. That’s why we didn’t observe wound contraction in mice directly, but our 3D collagen gel contraction assay alternatively proved that lumican can promote wound contraction.

Previous studies have shown that lumican exerts its functions predominantly through integrins [8], [24], [25], [31], [32]. But lumican-integrin interactions are different among different cell types. As reported, lumican promotes neutrophil migration by interaction with integrin β2 [8], while it inhibits melanoma cell migration through integrin α2β1 [25], [32]. In human umbilical vein endothelial cells, lumican blocks α2β1 integrin-mediated pro-angiogenic activity [24]. In this study we clearly demonstrated that lumican promotes dermal fibroblast contractility via integrin α2. The diversity of lumican functions through interaction with different integrins in different physiological and pathological conditions needs to be further investigated.

Tissue stiffness is implicated in many crucial physiological and pathological conditions, such as development, tumorigenesis, wound healing and fibrosis [33], [34],[35]. Tissue stiffness is primarily decided by two factors, thus ECM rigidity and contractile force generated by stromal cells [36], [37]. Lumican can bind with collagen fibril, the major ECM structure components, and regulate its assembly [38],which might affect ECM rigidity. Our studies also show that lumican can modulate mechanical properties of fibroblast, the primary stromal cell in skin, viscera organs [39] and many malignancy tumors [40], indicating that lumican might involve in regulation of mechanical aspects of microenvironment in these situations. So further exploration aiming at lumican’s roles in such fields might be interesting and fruitful.

Wound healing is a disturbing clinical situation with high morbidity and mortality [41], and satisfactory topical used drugs are still lacking [42]. Here we demonstrated that topical use of recombinant lumican protein can effectively promote mice skin wound healing, suggeting that lumican might be a potential bio-agent for human wound repair.
